# Prothrombotic hemostasis disturbances in patients with severe COVID-19: Individual daily data

**DOI:** 10.1016/j.dib.2020.106519

**Published:** 2020-11-10

**Authors:** Michaël Hardy, Isabelle Michaux, Sarah Lessire, Jonathan Douxfils, Jean-Michel Dogné, Marion Bareille, Geoffrey Horlait, Pierre Bulpa, Celine Chapelle, Silvy Laporte, Sophie Testa, Hugues Jacqmin, Thomas Lecompte, Alain Dive, François Mullier

**Affiliations:** a Université catholique de Louvain, CHU UCL Namur, Hematology Laboratory, Namur Thrombosis and Hemostasis Center (NTHC), Namur Research Institute for Life Sciences (NARILIS), Yvoir, Belgium; b Université catholique de Louvain, CHU UCL Namur, Anesthesiology Department, Namur Thrombosis and hemostasis Center (NTHC), Namur Research Institute for Life Sciences (NARILIS), Yvoir, Belgium; c Université catholique de Louvain, CHU UCL Namur, Department of Intensive Care, Yvoir, Belgium; d Department of Pharmacy, Namur Thrombosis and Hemostasis Center (NTHC), Namur Research Institute for Life Sciences (NARILIS), University of Namur, Namur, Belgium; e Qualiblood s.a., Namur, Belgium; f Unité de Recherche Clinique, Innovation, Pharmacologie, CHU Saint-Etienne, Hôpital Nord, F-42055 Saint-Etienne, France; g SAINBIOSE U1059, Université Jean Monnet, University Lyon, INSERM, F-CRIN INNOVTE Network, F-42023 Saint-Etienne, France; h Haemostasis and Thrombosis Center, Cremona Hospital, Cremona, Italy; i Département de Médecine, Hôpitaux Universitaires de Genève, service d'angiologie et d'hémostase et Faculté de Médecine, Geneva Platelet Group (GpG), Université de Genève, Geneva, Switzerland

**Keywords:** COVID-19, Intensive care unit, Thrombin generation, Fibrinolysis, Thrombosis, D-dimers, Hemostasis plasma proteins

## Abstract

This data article accompanies the manuscript entitled: “Prothrombotic Disturbances of hemostasis of Patients with Severe COVID-19: a Prospective Longitudinal Observational Cohort Study” submitted to *Thrombosis Research* by the same authors. We report temporal changes of plasma levels of an extended set of laboratory parameters during the ICU stay of the 21 COVID-19 patients included in the monocentre cohort: CRP, platelet count, prothrombin time; Clauss fibrinogen and clotting factors II, V and VIII levels, D-dimers, antithrombin activity, protein C, free protein S, total and free tissue factor pathway inhibitor, PAI-1 levels, von Willebrand factor antigen and activity, ADAMTS-13 (plasma levels); and of two integrative tests of coagulation (thrombin generation with ST Genesia) and fibrinolysis (global fibrinolytic capacity - GFC). Regarding hemostasis, we used double-centrifuged frozen citrated plasma prospectively collected after daily performance of usual coagulation tests. Demographic and clinical characteristics of patients and thrombotic and hemorrhagic complications were also collected from patient's electronic medical reports.

## Specifications Table

**Table:** 

Subject	Hematology
Specific subject area	Hemostasis, Coronavirus disease 2019
Type of data	Tables and Figures
How data were acquired	Blood samples prospectively collected daily from intensive care unit (ICU) patients admitted for coronavirus disease 2019 (COVID-19). Corresponding instruments and reagents of laboratory hematology for: platelet count, prothrombin time, Clauss fibrinogen and clotting factors II, V and VIII levels, D-dimers levels, PAI-1 levels, antithrombin activity, protein C activity, free protein S antigen, total and free tissue factor pathway inhibitor antigens, von Willebrand factor antigen and activity, ADAMTS-13 levels), thrombin generation, and global fibrinolytic capacity (GFC); and C-reactive protein.
Data format	Raw: Public repository. Analyzed: Tables and figures
Parameters for data collection	Laboratory data: Clinical laboratory tests that describe disturbances of hemostasis of ICU patients, severely affected with CoViD-19: primary hemostasis (platelet count, von Willebrand factor antigen and activity; ADAMTS-13 activity); coagulation (prothrombin time, Clauss fibrinogen, clotting factors II, V and VIII levels, in vitro thrombin potential), natural anticoagulants (antithrombin activity, protein C activity, free protein S antigen, total and free tissue factor pathway inhibitor antigens); and fibrinolysis (D-dimers levels, PAI-1 activity, global fibrinolytic capacity). Clinical data: complications of hemostasis disturbances (thrombosis and hemorrhages) and relevant data for characterization of the cohort (age; sex, BMI, ethnicity, comorbidities, APACHE II, SOFA scores and PaO_2_/FiO_2_ ratios at ICU admission, ICU stay duration, anticoagulation regimen, ICU length of stay, need for respiratory, cardiocirculatory or renal support; death).
Description of data collection	The following laboratory tests were performed with a STA-R Max (Diagnostica Stago, Asnières-sur-Seine) and reagents from Stago: prothrombin time (STA-NeoPTimal), Clauss fibrinogen (STA-Liquid FIB), clotting factor II (STA-NeoPTimal and STA – Deficient II), V (STA-NeoPTimal and STA – Deficient V) and VIII (STA-CK Prest and STA – Immunodef VIII), D-dimers (STA – LIATEST D-Di Plus), PAI-1 (STACHROM PAI-1), antithrombin (STA-Stachrom ATIII), protein C activity (STA – Stachrom Protein C), free protein S antigen (STA-LIATEST Free Protein S), total (Asserachrom total TFPI) and free TFPI (Asserachrom free TFPI), heparin anti-Xa activity (STA - Liquid anti-Xa), von Willebrand factor antigen (STA – LIATEST VWF:Ag). CRP levels were measured on a Vitros 5600 Integrated System with CRP Gold Latex reagents (DiAgam, Ghislenghien, Belgium) and platelet count on a Sysmex
	XN-20 analyzer with Cellpack reagent (Sysmex). Thrombin generation was measured on a ST Genesia with STG-ThromboScreen reagent (Stago) after neutralizing heparin with hexadimethrine bromide (25 μg/mL; polybrene, Sigma Aldrich, Saint-Louis, United States). Global fibrinolytic capacity was measured using the Lysis Timer instrument with dedicated reagents (Hyphen Biomed). ADAMTS13 activity was measured using the Technozym® ADAMTS-13 Activity ELISA kit (Technozym, Technoclone, Vienna, Austria). Von Willebrand activity was measured on an AcuStar analyser with HemosIL AcuStar VWF:RCo reagent (Instrumentation Laboratory). Laboratory data were retrieved from laboratory files. Clinical data were retrieved from patients’ medical charts.
Data source location	Institution: CHU UCL Namur – Godinne site. City: Yvoir. Country: Belgium.
Data accessibility	Repository name: Mendeley Data Data identification number: 10.17632/g7x5nvj9d2.1 Direct URL to data: https://dx.doi.org/10.17632/g7x5nvj9d2.1
Related research article	M. Hardy, I. Michaux, S. Lessire, J. Douxfils, J.-M. Dogné, M. Bareille, G. Horlait, P. Bulpa, C. Chapelle, S. Laporte, S. Testa, H. Jacqmin, T. Lecompte, A. Dive and F. Mullier. Prothrombotic hemostasis Disturbances in Patients with Severe COVID-19: a Prospective Longitudinal Observational Cohort Study. Thromb Res. 2020; 197:20-23. [1]

## Value of the Data

• The data reported with individual time-courses during the ICU stay show the variability of hemostasis parameters over time and between individuals, suggesting varying thrombotic risks and the need for individualization of thrombotic prophylaxis, with frequent reassessments.
• They can benefit to all physicians and scientists dealing with COVID-19.
• These data will be helpful to design further prospective studies focusing on COVID-19 hemostasis disorders: which parameters to measure and at which frequency.

## 1 Data Description

Demographic and clinical characteristics of observed ICU patients are shown in Table 1. Values correspond to median (with interquartile and min-max ranges) for quantitative data and to number (percent) for qualitative data.

**Table 1:** Demographic and clinical characteristics of observed patients.

	All patients(*n*=21)
Age (years), median (IQR)	60 [57–64] (48–74)
Male gender, n (total)	18 (21)
BMI (kg/m²), median (IQR)	30 [27–32] (21–42)
Caucasian ethnicity, n (total)	18 (21)
Obesity (BMI ≥30 kg/m²), n (total)	11 (21)
Hypertension, n (total)	11 (21)
COPD, n (total)	4 (21)
Asthma, n (total)	3 (21)
CAD, n (total)	3 (21)
On arrival at the ICU	
APACHE II, median (IQR)	14 [12–20] (9–22)
SOFA score, median (IQR)	6 [4–8] (3–12)
Time-interval between symptoms onset and ICU admission, median (IQR)	9 [8–11] (4–15)
PAFI, median (IQR)	91 [67–135] (44–260)
During ICU stay	
ICU stay duration, median (IQR)	15 (7–26) (5–28)
Patients having received UFH^a^, n (total)	13 (21)
UFH daily dose (IU/kg/day), median (IQR)	441 [338–587] (0–800)
Patients having received LMWH^a^, n (total)	19 (21)
LMWH daily dose (IU/kg/day), median (IQR)	100 [53–149] (0–230)
Non-invasive ventilation, n (tot)	3 (21)
Invasive ventilation support, n (tot)	19 (21)
ECMO, n (tot)	5 (21)
Vasopressor use, n (tot)	16 (21)
New renal replacement therapy, n (tot)	6 (21)
Thrombotic complications^b^	10 (21)
Major bleeding according to the ISTH^b^	6 (21)
Death, n (tot)	3 (21)
Discharged from ICU at the end of observation period, n (tot)	9 (21)

a Patients may have received UFH or LMWH at different periods of ICU stay.

b See the companion paper for the descriptions of the events. IQR, interquartile range; BMI, body mass index; COPD, chronic obstructive pulmonary disease; CAD, coronary artery disease; ICU, intensive care unit; IQR, interquartile range; APACHE II score, acute physiology and chronic health disease classification system II; SOFA, sepsis-related organ failure assessment; PAFI, PaO_2_/FiO_2_ ratio; UFH, unfractionated heparin; LWMH, low molecular weight heparin; ECMO, extracorporeal membrane oxygenation; ICU, intensive care unit.

Baseline (D0) was defined as ICU admission (in Namur or elsewhere; 11 patients were transferred from the ICU of another Belgian hospital), but the laboratory-monitoring period was restricted to the Namur ICU stay. Tests on D0 were often missing due to delays in patients’ inclusion.

Table 2 represents the changes over time of hemostasis parameters along ICU stay of 21 severe COVID-19 patients. Observation period has been arbitrarily subdivided into three time-intervals of 10 days starting from D1. For each patient and time-interval, parameters medians were calculated. Medians and interquartile ranges of patient's medians are presented for the three time-intervals. Minimum and maximum values observed are also represented. D-dimers plasma levels are expressed in fibrinogen equivalent units (FEU) and ‘reference ranges’ depicted correspond to DIC thresholds according to the ISTH definition with the reagents we used [2].

**Table 2:** Changes over time of hemostasis parameters along intensive care unit stay.

Variable and reference ranges	D1–D10 (*n* = 18)	D11–D20 (*n* = 15)	D21–D30 (*n* = 9)
CRP (mg/dL) <5	211 [160–267] (9–485)	97 [63–126] (2–451)	95 [64–114] (14–256)
**Primary hemostasis**			
Platelet count (10^9^/L) 150 to 450	246 [177–387] (73–701)	337 [187–454] (89–672)	252 [153–412] (87–583)
vWF antigen (%) 50 to 160	438 [357–534] (204–803)	476 [380–548] (161–846)	475 [424–702] (211–795)
vWF activity (%) 46 to 176	294 [228–452] (151–845)	344 [280–400] (107–927)	429 [383–676] (152–779)
ADAMTS-13 (IU/mL) 0.4 to 1.3	0.61 [0.40–0.65] (0.13–0.86)	0.45 [0.39–0.61] (0.24–0.83)	0.45 [0.43–0.50] (0.41–0.62)
**Coagulation**			
Prothrombin time (%) 75 to 100	81 [75–89] (28–100)	80 [77–87] (51–100)	80 [78–84] (62–100)
Fibrinogen (mg/dL) 200 to 400	722 [588–820] (177–1122)	680 [555–723] (64–985)	631 [592–682] (198–851)
Factor II (%) 70 to 120	82 [74–97] (34–126)	88 [70–96] (30–113)	94 [79–103] (37–119)
Factor V (%) 70 to 120	127 [91–158] (21–218)	140 [112–175] (64–245)	126 [114–145] (42–299)
Factor VIII (%) 60 to 150	292 [239–317] (100–522)	326 [263–354] (99–603)	333 [268–404] (128–576)
AT (%) 80 to 120	91 [68–100] (33–131)	82 [71–105] (51–130)	92 [78–94] (53–107)
Protein C activity (%) 70–120	85 [78–120] (46–169)	113 [105–117] (51–178)	107 [98–158] (90–187)
Free protein S antigen (%) Men: 70 to 148 Women: 50 to 134	80 [65–124] (39–150)	114 [88–129] (44–150)	110 [96–149] (45–150)
Total TFPI (ng/mL) 20.4 to 142	107 [82–168] (60–292)	98 [90–158] (57–396)	128 [92–140] (50–186)
Free TFPI (ng/mL) 0.4 to 19.6	42 [28–75] (9–173)	32 [22–83] (12–175)	38 [23–57] (14–102)
Thrombin generation lag time (ratio) 1.1 to 1.3	2.0 [1.5–2.4] (1.0–10.3)	2.2 [1.7–3.0] (1.1–9.9)	2.5 [1.9–3.3] (1.4–7.6)
Thrombin generation time to peak (ratio) 1.2 to 1.3	1.7 [1.4–2.0] (1.0–6.5)	1.8 [1.5–2.4] (1.0–6.5)	2.0 [1.7–2.5] (1.1–5.1)
Thrombin generation peak height (%) 45 to 66	78 [53–105] (13–139)	67 [48–78] (21–137)	54 [42–73] (19–174)
Thrombin generation ETP (%) 59 to 80	111 [70–139] (23–208)	81 [66–89] (37–210)	73 [56–95] (31–158)
**Fibrinolysis**			
D-dimers (ng/mL) ISTH criterion for DIC, ≥3500 (2 pts) or ≥11,100 (3 pts) [2]	4860 [2336–12,260] (750–20,000)	3425 [2557–4710] (540–13,750)	3120 [1960–4420] (800–16,430)
PAI-1 (AU/mL) <16	23.6 [20.3–25.4] (2.3–58.7)	19.5 [16.5–28.8] (1.6–52.0)	15.6 [13.9–57.2] (6.8–52.2)
GFC (min) 30 to 60	57 [53–64] (31–240)	55 [49–69] (34–240)	57 [44–73] (37–240)

CRP, C-reactive protein; vWF antigen, von Willebrand factor antigen; vWF activity, von Willebrand factor activity, ristocetin cofactor; ADAMTS-13 (also known as von Willebrand factor cleaving protease); TFPI, tissue factor pathway inhibitor; DIC, disseminated intravascular coagulopathy; t-PA, tissue-type plasminogen activator; PAI-1, plasminogen activator inhibitor; AU, arbitrary units; GFC, global fibrinolysis capacity (Lysis Timer instrument); ETP, endogenous thrombin potential.

Results of ST Genesia TGA are relative to reference plasma and expressed as ratios (temporal parameters) or percentages (thrombin concentration-related parameters).

The figures represent the changes over time of measured hemostasis parameters during the ICU stay of each of the 21 patients. Blue lines represent the reference range locally determined, or previously published under similar analytical conditions, or according to the manufacturer's (see figure legends). Stars represent the follow-up period of the patients; orange stars represent the day of diagnosis of a thrombotic complication (which might be delayed form the actual onset) (Fig. 1, Fig. 2, Fig. 3, Fig. 4, Fig. 5, Fig. 6, Fig. 7, Fig. 8, Fig. 9, Fig. 10, Fig. 11, Fig. 12, Fig. 13, Fig. 14, Fig. 15, Fig. 16, Fig. 17, Fig. 18, Fig. 19, Fig. 20, Fig. 21, Fig. 22).

**Fig. 1:**
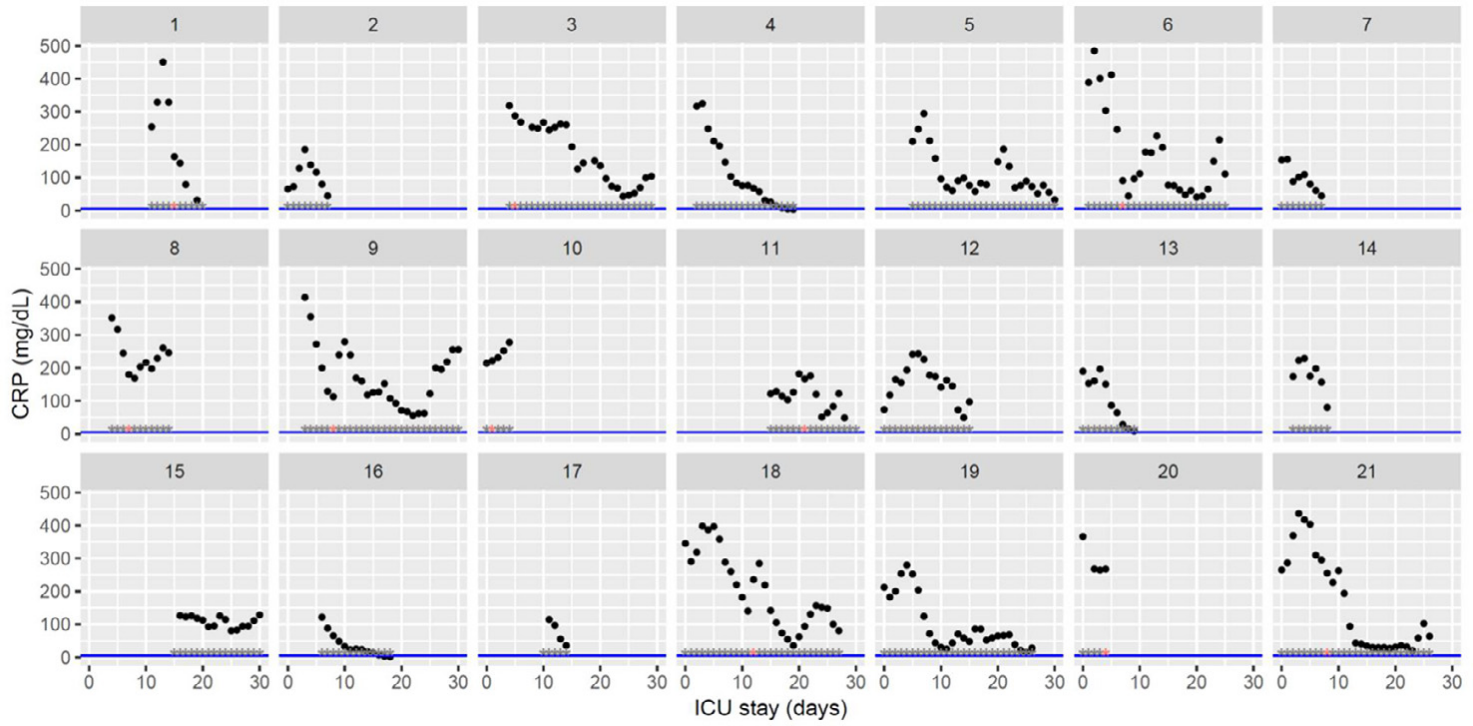
Temporal changes in C-reactive protein plasma levels during Namur ICU stay for the 21 patients. Each point represents the result of the test of the day. The blue line represents the reference range according to the manufacturer (5 mg/dL). Grey stars represent the inclusion period and orange stars the day of thrombosis diagnosis.(For interpretation of the references to color in this figure legend, the reader is referred to the web version of this article.)

**Fig. 2:**
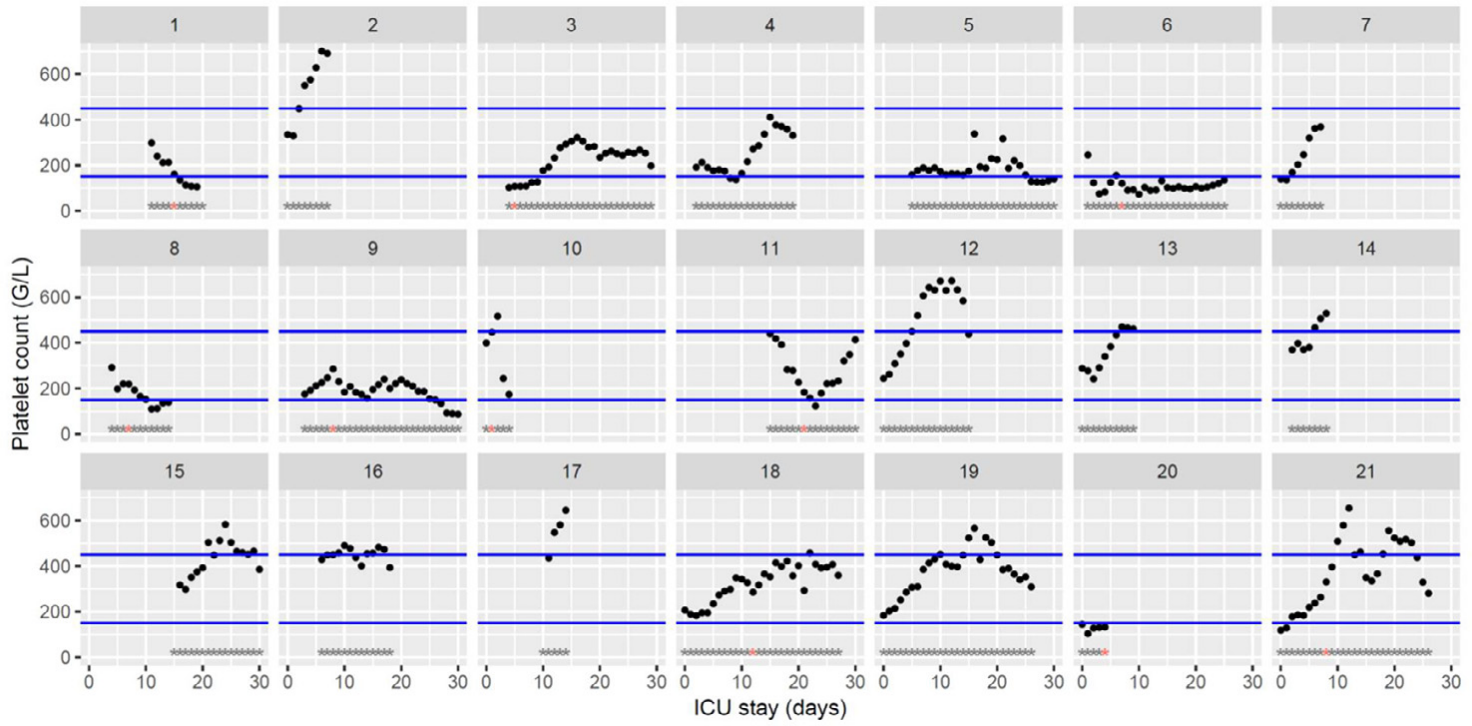
Temporal changes in platelet count during Namur ICU stay for the 21 patients. Each point represents the result of the test of the day. Blue lines represent the reference range (150–450 × 10^9^/L). Grey stars represent the inclusion period and orange stars the day of thrombosis diagnosis.(For interpretation of the references to color in this figure legend, the reader is referred to the web version of this article.)

**Fig. 3:**
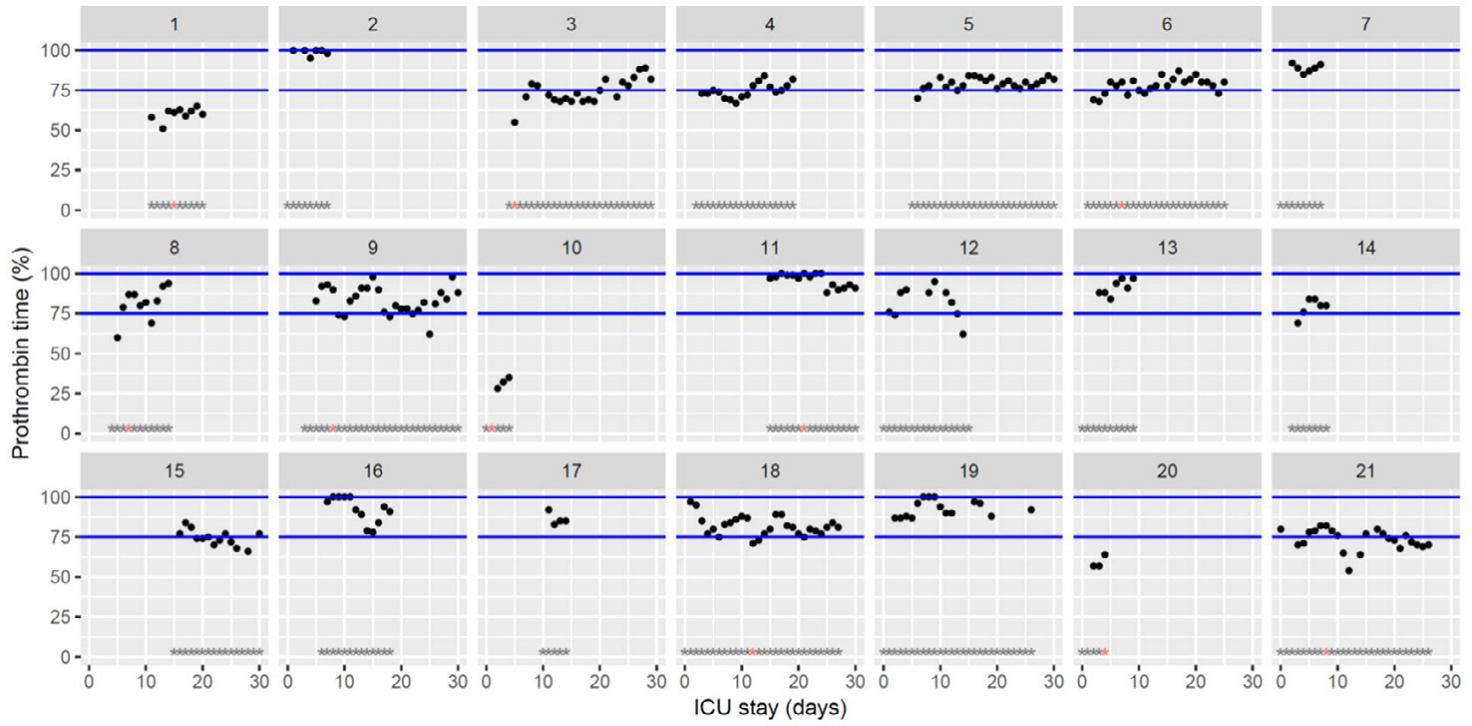
Temporal changes in prothrombin time (expressed as percentage activity) during Namur ICU stay for the 21 patients. Each point represents the result of the test of the day. Blue lines represent the reference range locally determined (75–100%). Grey stars represent the inclusion period and orange stars the day of thrombosis diagnosis.(For interpretation of the references to color in this figure legend, the reader is referred to the web version of this article.)

**Fig. 4:**
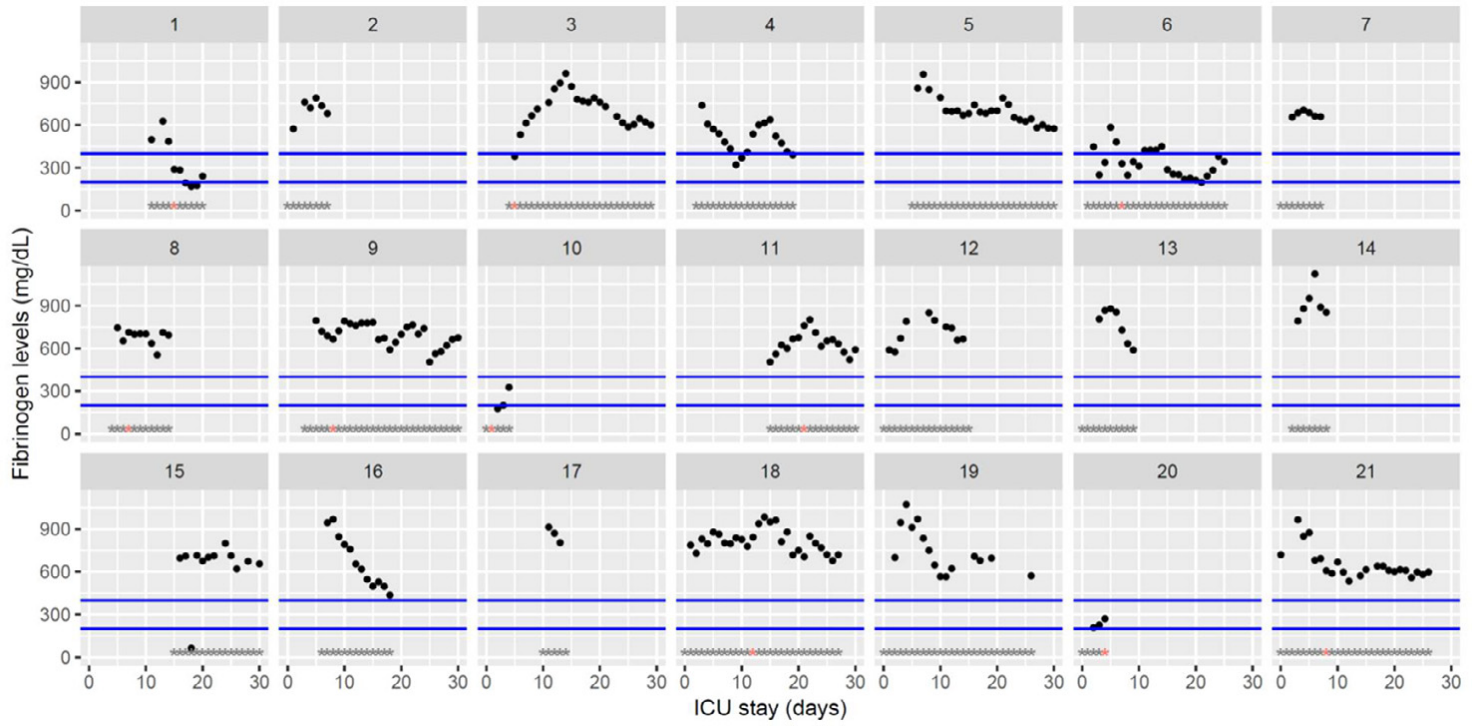
Temporal changes in Clauss fibrinogen during Namur ICU stay for the 21 patients. Each point represents the result of the test of the day. Blue lines represent the reference range according to the manufacturer (200–400 mg/dL). Grey stars represent the inclusion period and orange stars the day of thrombosis diagnosis.(For interpretation of the references to color in this figure legend, the reader is referred to the web version of this article.)

**Fig. 5:**
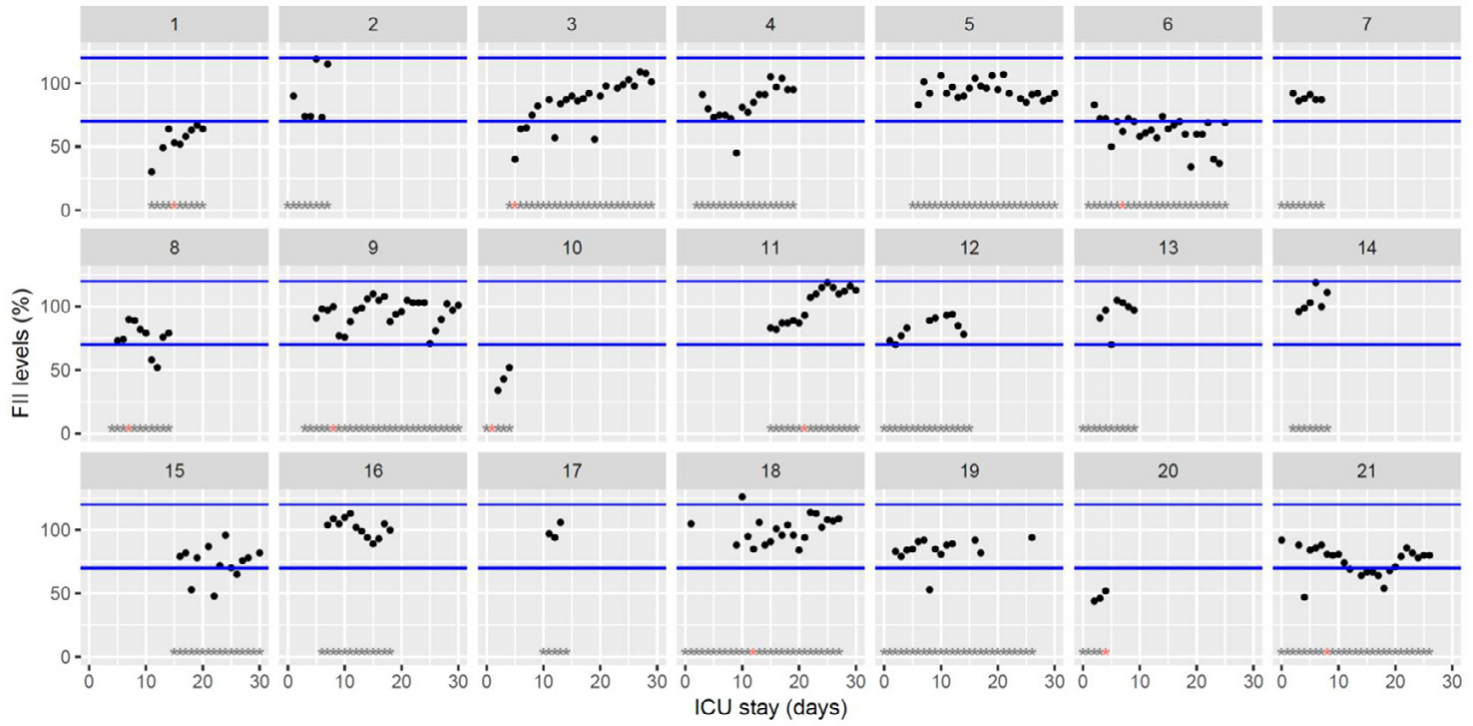
Temporal changes in clotting factor II levels during Namur ICU stay for the 21 patients. Each point represents the result of the test of the day. Blue lines represent the reference range according to the manufacturer (70–120%). Grey stars represent the inclusion period and orange stars the day of thrombosis diagnosis.(For interpretation of the references to color in this figure legend, the reader is referred to the web version of this article.)

**Fig. 6:**
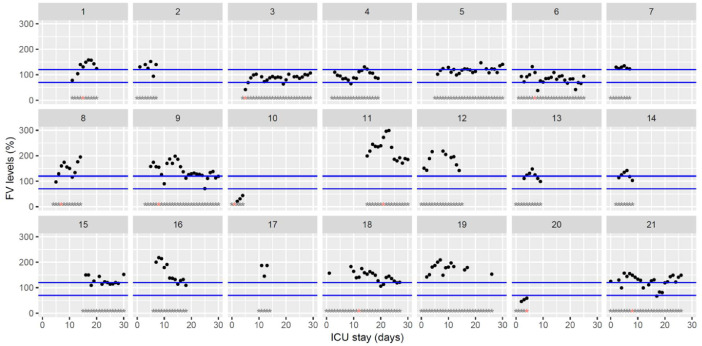
Temporal changes in clotting factor V levels during Namur ICU stay for the 21 patients. Each point represents the result of the test of the day. Blue lines represent the reference range according to the manufacturer (70–120%). Grey stars represent the inclusion period and orange stars the day of thrombosis diagnosis.(For interpretation of the references to color in this figure legend, the reader is referred to the web version of this article.)

**Fig. 7:**
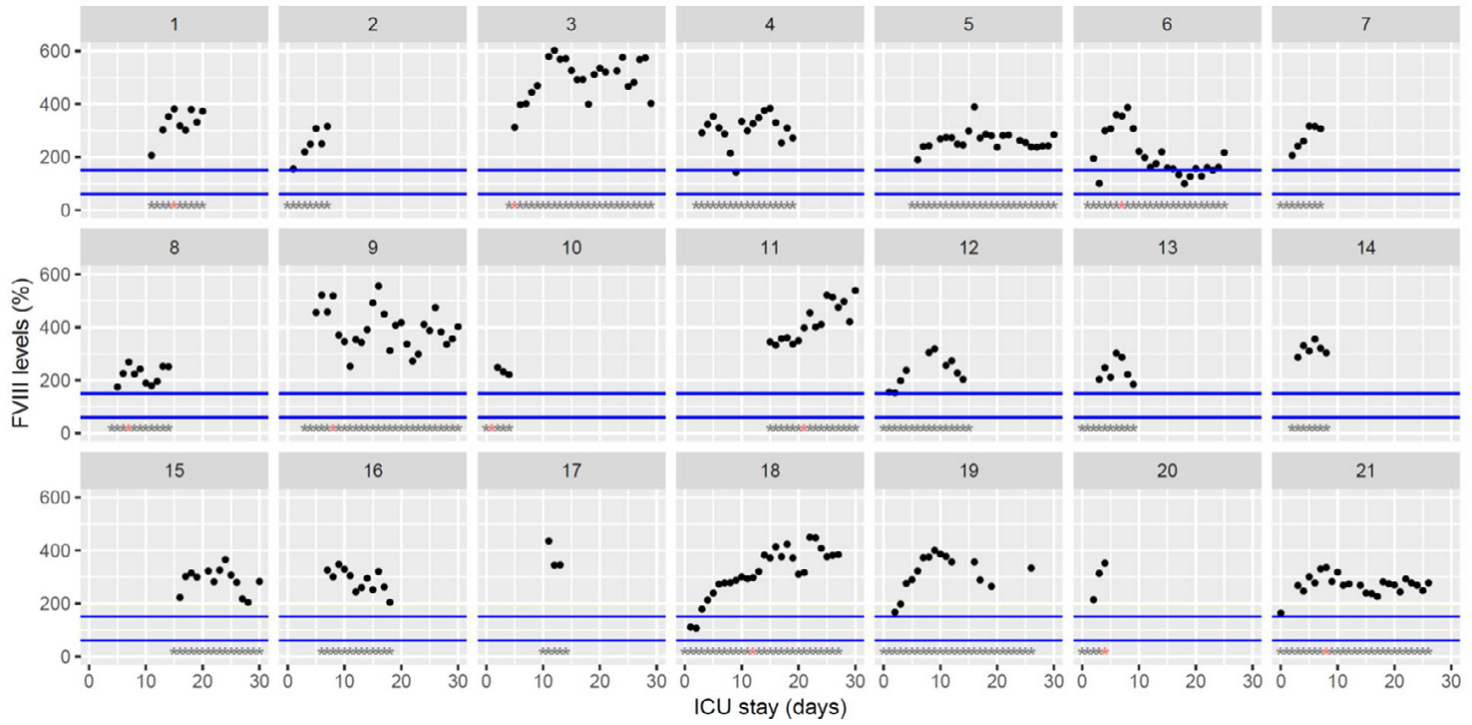
Temporal changes in clotting factor VIII levels during Namur ICU stay for the 21 patients. Each point represents the result of the test of the day. Blue lines represent the reference range according to the manufacturer (60–150%). Grey stars represent the inclusion period and orange stars the day of thrombosis diagnosis.(For interpretation of the references to color in this figure legend, the reader is referred to the web version of this article.)

**Fig. 8:**
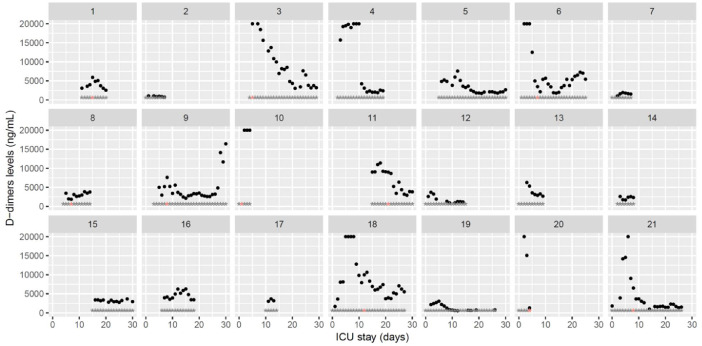
Temporal changes in D-dimers plasma levels during Namur ICU stay for the 21 patients. Each point represents the result of the test of the day. Grey stars represent the inclusion period and orange stars the day of thrombosis diagnosis.(For interpretation of the references to color in this figure legend, the reader is referred to the web version of this article.)

**Fig. 9:**
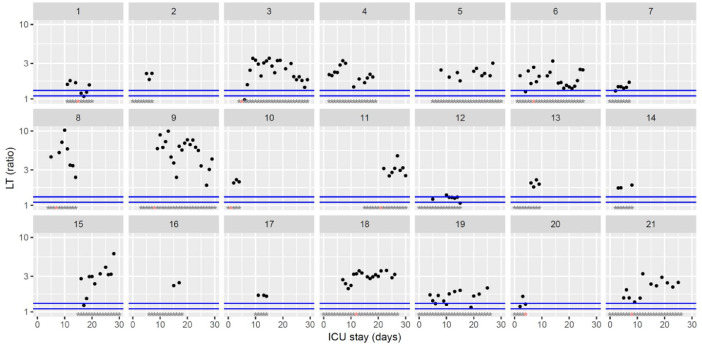
Temporal changes in thrombin generation (ST Genesia with ThromboScreen reagent) lag time (LT) normalized using a reference plasma (provided with ThromboScreen reagent) during Namur ICU stay for the 21 patients. Each point represents the result of the test of the day. Blue lines represent the reference range according to Calzavarini et al (1.1–1.3) [3]. Grey stars represent the inclusion period and orange stars the day of thrombosis diagnosis. The ordinate is represented using a logarithmic scale.(For interpretation of the references to color in this figure legend, the reader is referred to the web version of this article.)

**Fig. 10:**
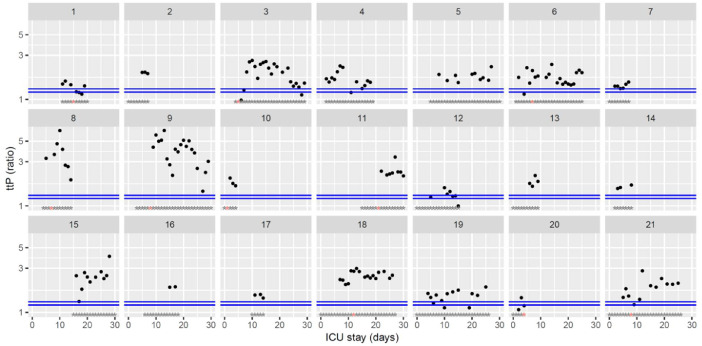
Temporal changes in thrombin generation (ST Genesia with ThromboScreen reagent) time to peak (ttP) normalized using a reference plasma (provided with ThromboScreen reagent) during Namur ICU stay for the 21 patients. Each point represents the result of the test of the day. Blue lines represent the reference range according to Calzavarini et al. (1.2–1.3) [3]. Grey stars represent the inclusion period and orange stars the day of thrombosis diagnosis. The ordinate is represented using a logarithmic scale.(For interpretation of the references to color in this figure legend, the reader is referred to the web version of this article.)

**Fig. 11:**
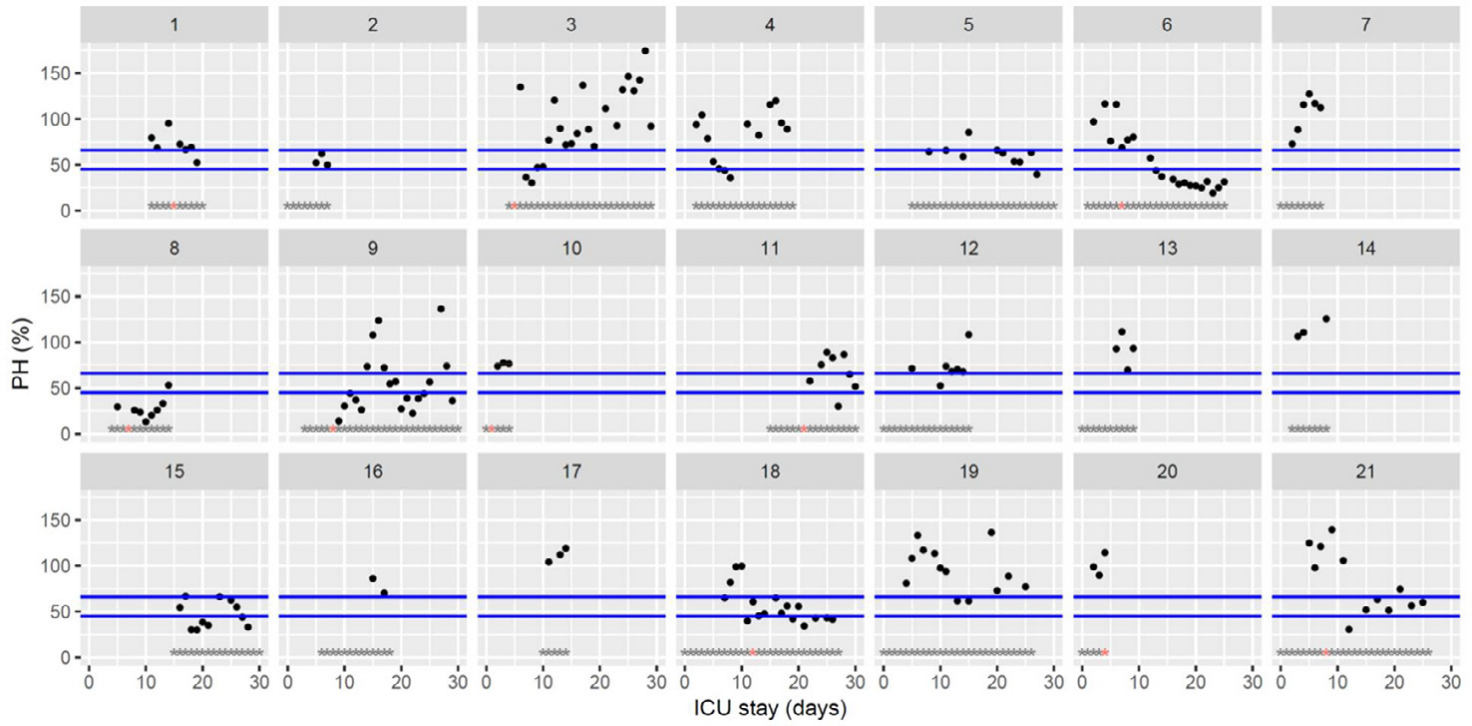
Temporal changes in thrombin generation (ST Genesia with ThromboScreen reagent) peak height (pH) normalized using a reference plasma (provided with ThromboScreen reagent) during Namur ICU stay for the 21 patients. Each point represents the result of the test of the day. Blue lines represent the reference range according to Calzavarini et al. (45–66%) [3]. Grey stars represent the inclusion period and orange stars the day of thrombosis diagnosis.(For interpretation of the references to color in this figure legend, the reader is referred to the web version of this article.)

**Fig. 12:**
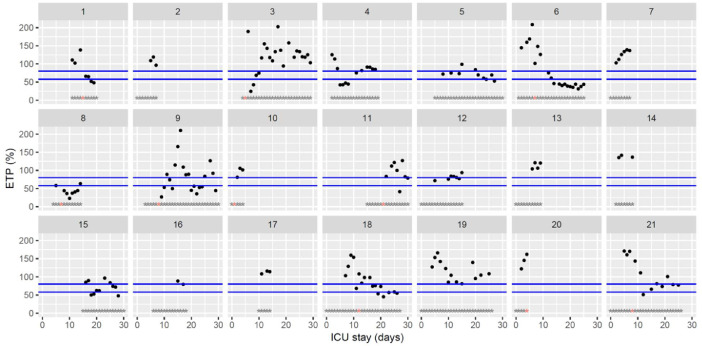
Temporal changes in thrombin generation (ST Genesia with ThromboScreen reagent) endogenous thrombin potential (ETP) normalized using a reference plasma (provided with ThromboScreen reagent) during Namur ICU stay for the 21 patients. Each point represents the result of the test of the day. Blue lines represent the reference range according to Calzavarini et al (59–80%) [3]. Grey stars represent the inclusion period and orange stars the day of thrombosis diagnosis.(For interpretation of the references to color in this figure legend, the reader is referred to the web version of this article.)

**Fig. 13:**
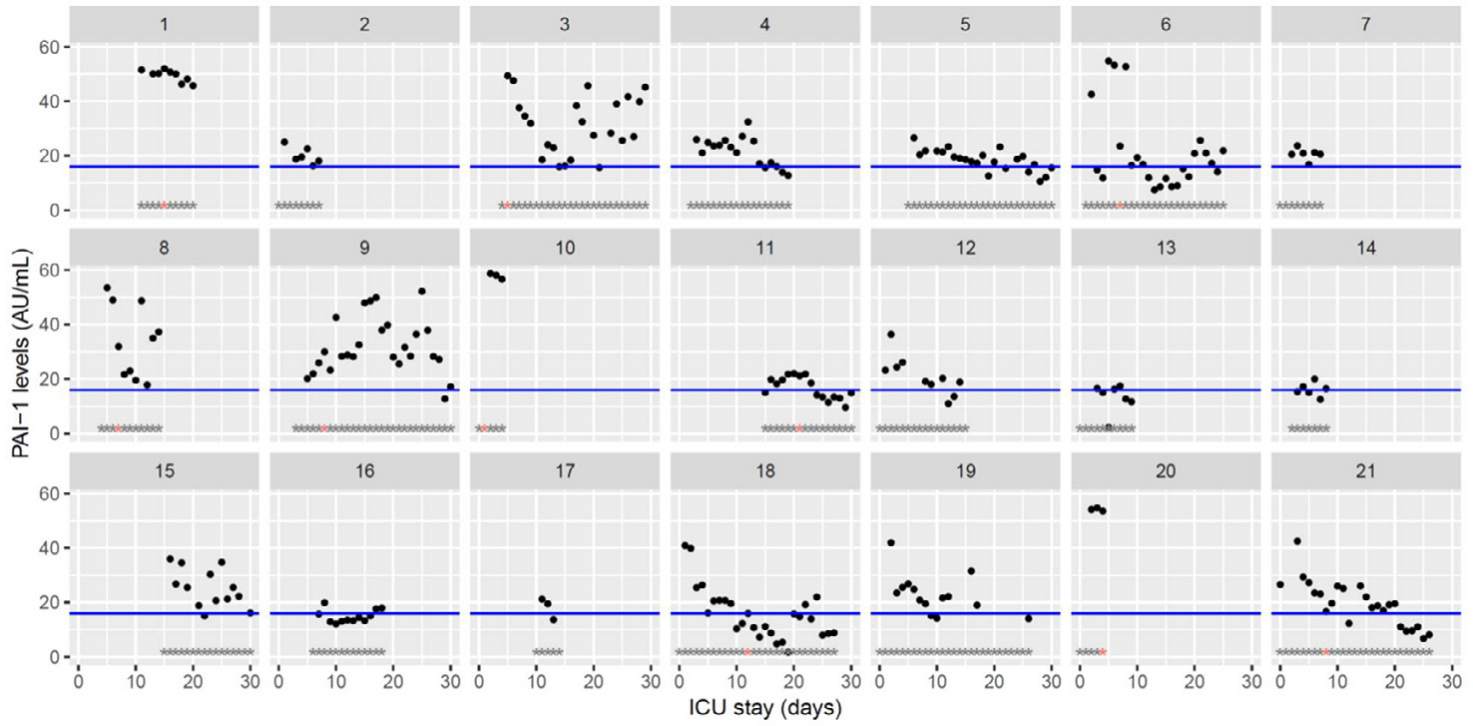
Temporal changes in PAI-1 activity during Namur ICU stay for the 21 patients. Each point represents the result of the test of the day. The blue line represents the reference range according to the manufacturer (<16AU/mL). Grey stars represent the inclusion period and orange stars the day of thrombosis diagnosis.(For interpretation of the references to color in this figure legend, the reader is referred to the web version of this article.)

**Fig. 14:**
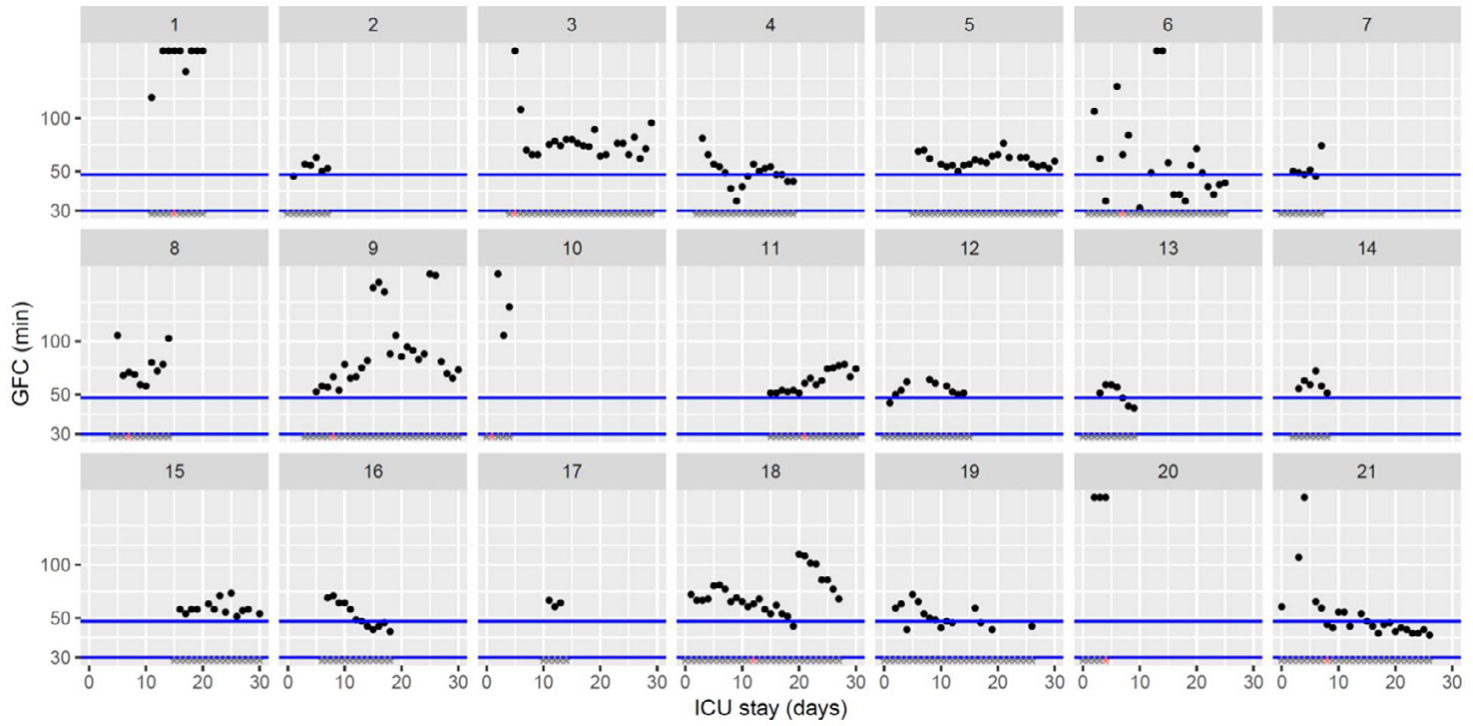
Temporal changes in global fibrinolytic capacity (GFC) measured with the Lysis Timer instrument during Namur ICU stay for the 21 patients. Each point represents the result of the test of the day. Blue lines represent the reference range locally determined (30–48 min). Grey stars represent the inclusion period and orange stars the day of thrombosis diagnosis. The ordinate is represented using a logarithmic scale.(For interpretation of the references to color in this figure legend, the reader is referred to the web version of this article.)

**Fig. 15:**
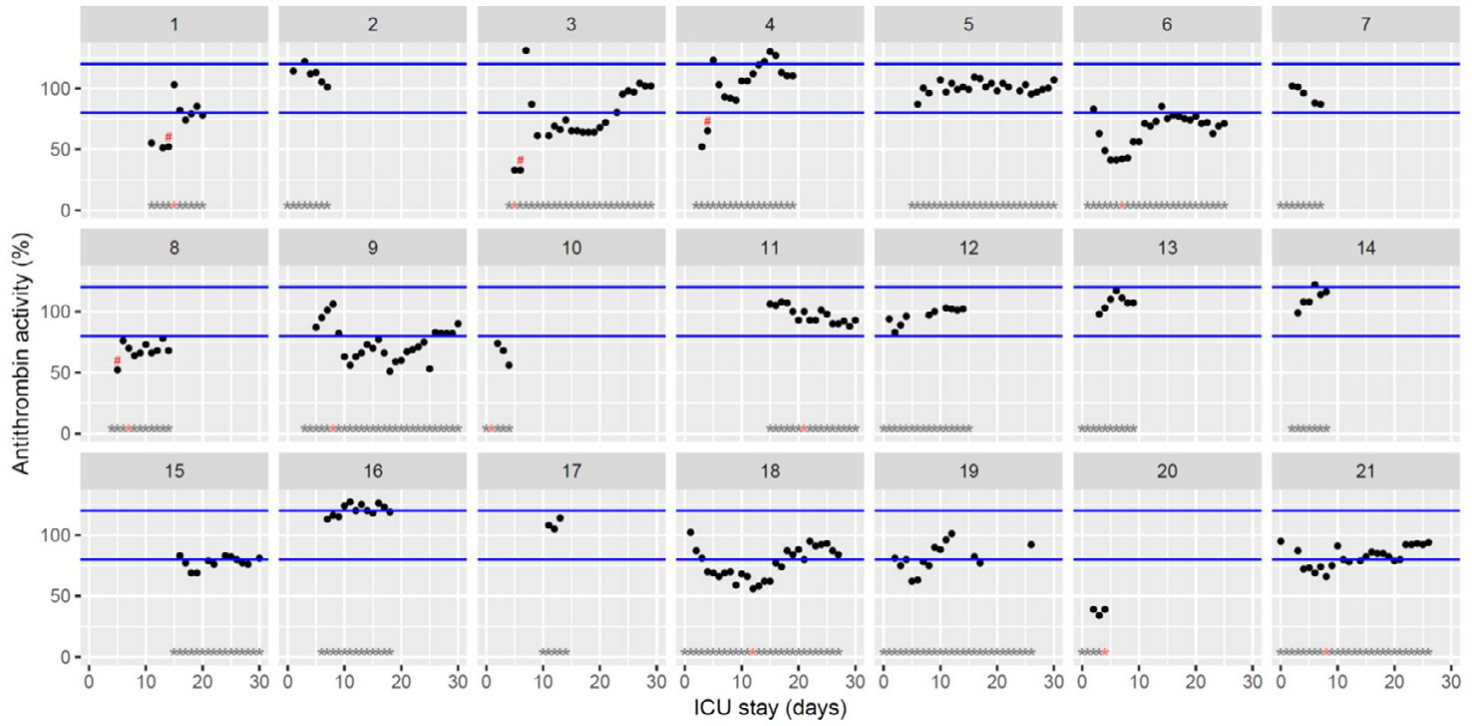
Temporal changes in antithrombin activity during Namur ICU stay for the 21 patients. Each point represents the result of the test of the day. Blue lined represent the reference range according to the manufacturer (80–120%). Grey stars represent the inclusion period and orange stars the day of thrombosis diagnosis. Exogenous antithrombin supplementations are represented by red hashes.(For interpretation of the references to color in this figure legend, the reader is referred to the web version of this article.)

**Fig. 16:**
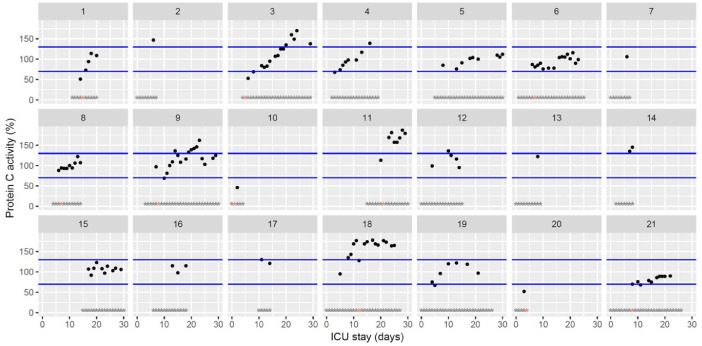
Temporal changes in protein C activity during Namur ICU stay for the 21 patients. Each point represents the result of the test of the day. Blue lines represent the reference range according to the manufacturer (70–130%). Grey stars represent the inclusion period and orange stars the day of thrombosis diagnosis.(For interpretation of the references to color in this figure legend, the reader is referred to the web version of this article.)

**Fig. 17:**
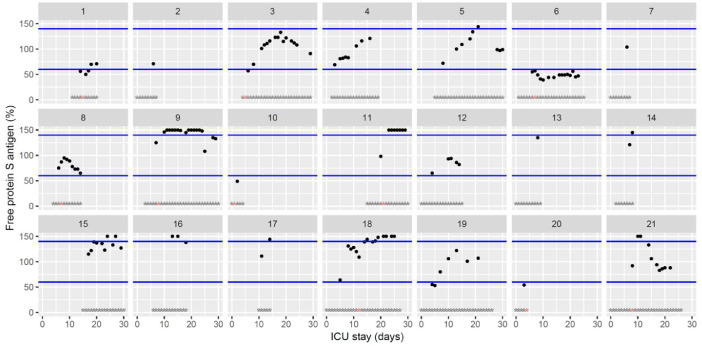
Temporal changes in free protein S levels during Namur ICU stay for the 21 patients. Each point represents the result of the test of the day. Blue lines represent the reference range according to the manufacturer (60–140%). Grey stars represent the inclusion period and orange stars the day of thrombosis diagnosis.(For interpretation of the references to color in this figure legend, the reader is referred to the web version of this article.)

**Fig. 18:**
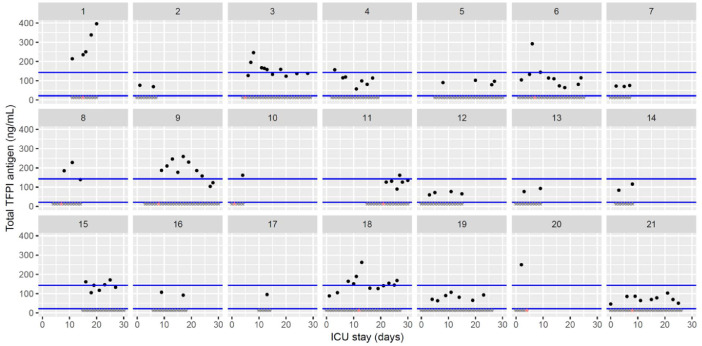
Temporal changes in total tissue factor pathway inhibitor (TFPI) levels during Namur ICU stay for the 21 patients. Each point represents the result of the test of the day. Blue lines represent the reference range according to the manufacturer (21.3–142.9 ng/mL). Grey stars represent the inclusion period and orange stars the day of thrombosis diagnosis.(For interpretation of the references to color in this figure legend, the reader is referred to the web version of this article.)

**Fig. 19:**
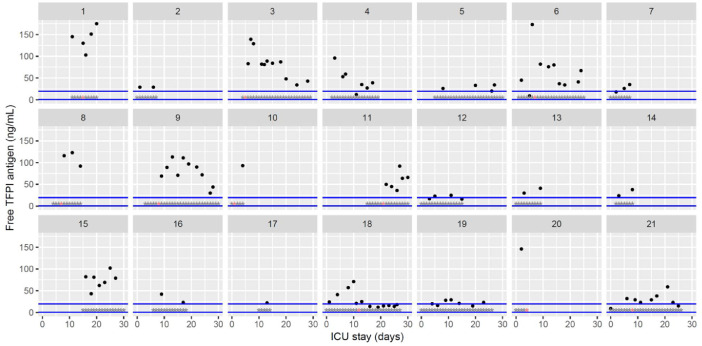
Temporal changes in free tissue factor pathway inhibitor (TFPI) levels during Namur ICU stay for the 21 patients. Each point represents the result of the test of the day. Blue lines represent the reference range according to the manufacturer (0.4–19.6 ng/mL). Grey stars represent the inclusion period and orange stars the day of thrombosis diagnosis.(For interpretation of the references to color in this figure legend, the reader is referred to the web version of this article.)

**Fig. 20:**
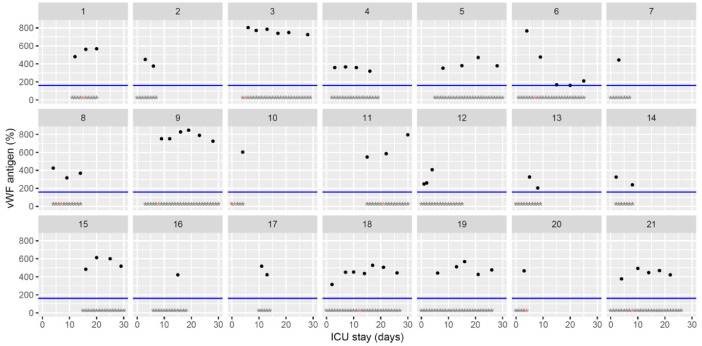
Temporal changes in von Willebrand factor (vWF) antigen levels during Namur ICU stay for the 21 patients. Each point represents the result of the test of the day. The blue line represents the reference range according to the manufacturer (< 160%). Grey stars represent the inclusion period and orange stars the day of thrombosis diagnosis.(For interpretation of the references to color in this figure legend, the reader is referred to the web version of this article.)

**Fig. 21:**
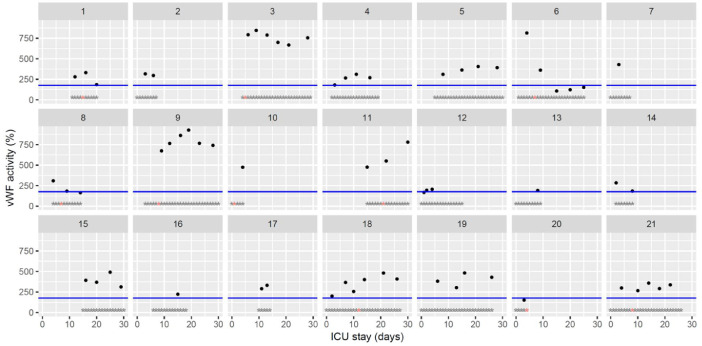
Temporal changes in von Willebrand factor (vWF) activity (ristocetin cofactor) during Namur ICU stay for the 21 patients. Each point represents the result of the test of the day. The blue line represents the reference range according to the manufacturer (< 176%). Grey stars represent the inclusion period and orange stars the day of thrombosis diagnosis.(For interpretation of the references to color in this figure legend, the reader is referred to the web version of this article.)

**Fig. 22:**
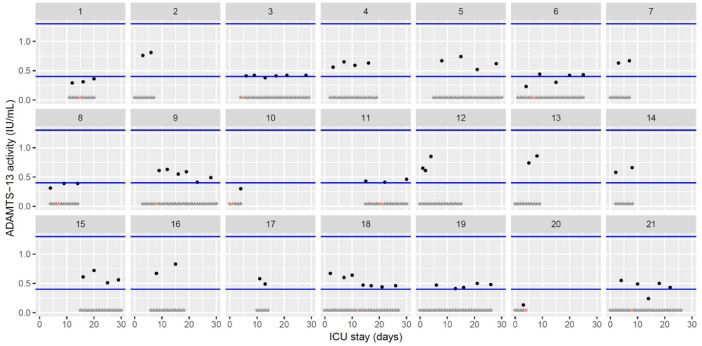
Temporal changes ADAMTS-13 activity during Namur ICU stay for the 21 patients. Each point represents the result of the test of the day. Blue lines represent the reference range according to the manufacturer (0.4–1.3IU/mL). Grey stars represent the inclusion period and orange stars the day of thrombosis diagnosis.(For interpretation of the references to color in this figure legend, the reader is referred to the web version of this article.)

## 2 Experimental Design, Materials and Methods

### 2.1 Setting

CHU UCL Namur (Godinne site, Yvoir, Belgium), a tertiary academic hospital.

### 2.2 Patients

All patients admitted to the intensive care unit (ICU) of the CHU UCL Namur for an RT-PCR confirmed severe acute respiratory syndrome coronavirus 2 (SARS-CoV2) infection from March 27 to April 24, 2020 were considered for inclusion. Twenty-one patients were finally included, one patient being excluded because of major therapeutic limitation (i.e. refusal of tracheal intubation).

### 2.3 Clinical management

Patients’ characteristics were collected at first ICU admission. Patients were managed according to local standard of care. Anticoagulation guidelines from the Groupe d'Intérêt en Hémostase Périopératoire (GIHP) were implemented in Namur from April 2, 2020 [4]. Patients were screened for deep vein thrombosis (DVT) within a week after Namur ICU admission, and then once a week unless a thrombotic event occurred. Pulmonary embolism (as a matter of fact could be in situ arterial thrombosis) was diagnosed directly by contract CT scan or indirectly by transesophageal ultrasonography (for unstable patients that cannot be transferred safely to the Radiology Department). Bleeding events were defined as minor or major according to ISTH definitions [5].

### 2.4 Blood samples

Blood samples were collected from the arterial line as part of clinical patients’ care and at least once a day around 4a.m. Serum was prepared from plastic tubes containing alumina silicate as coagulation activator (Vacuette, Greiner Bio One, Kremsmünster, Austria), whole blood was collected in K2-ethylenediaminetetraceatic acid (EDTA) tubes (Vacuette, Greiner Bio One) and plasma was prepared from 109 mM citrate tubes (Vacuette, Greiner Bio One) using double centrifugation (1500 g, 15 min, room temperature). Plasma samples were frozen at -80 °C and thawed at 37 °C for 5 min immediately before analysis.

### 2.5 Laboratory tests

Laboratory tests were performed on 4a.m. samples whenever possible or on the temporally closest samples.

CRP levels were measured on a Vitros 5600 Integrated System (Ortho Clinical Diagnostics, Belgium) with CRP Gold Latex reagents (DiAgam, Ghislenghien, Belgium) and platelet count on a Sysmex XN-20 analyzer with Cellpack reagent (Sysmex Corporation, Kobe, Japan).

The following laboratory tests were performed with a STA-R Max (Stago, Asnières-sur-Seine) and reagents from Stago: prothrombin time (STA-NeoPTimal; expressed as percentage [6]), Clauss fibrinogen (STA-Liquid FIB), clotting factor II (STA-NeoPTimal and STA – Deficient II), V (STA-NeoPTimal and STA – Deficient V) and VIII (STA-CK Prest and STA – Immunodef VIII), antithrombin (STA-Stachrom ATIII), D-dimers (STA – LIATEST D-Di Plus), PAI-1 (STACHROM PAI-1), von Willebrand factor antigen (STA – LIATEST VWF:Ag), protein C activity (STA – Stachrom Protein C), free protein S antigen (STA-LIATEST Free Protein S), total (Asserachrom total TFPI) and free TFPI (Asserachrom free TFPI).

Thrombin generation was measured with ST Genesia and STG-ThromboScreen reagent (Stago) after neutralizing heparin with hexadimethrine bromide (25 μg/mL; polybrene, Sigma Aldrich, Saint-Louis, United States) [3,7]. Global fibrinolytic capacity was measured using the Lysis Timer instrument (Hyphen Biomed, Neuville-sur-Oise, France; SD Innovation, Frouard, France, respectively) with dedicated reagents (Hyphen Biomed) [8]. ADAMTS-13 activity was measured using the Technozym® ADAMTS-13 Activity ELISA kit (Technozym, Technoclone, Vienna, Austria). Von Willebrand activity was measured with an AcuStar analyser (Instrumentation Laboratory, Bedford, USA) and HemosIL AcuStar VWF:RCo reagent (Instrumentation Laboratory).

Most analyses were intended to be performed once a day whenever possible. Some analyses were purposely performed only every 5 days (i.e. vWF antigen and activity, ADAMTS-13, total and free TFPI, tissue-type plasminogen activator) or every other day (protein C activity, free protein S antigen). The percentage of days with available data for the whole observation period is represented in Table 3.

### 2.6 Analysis

Figures were performed in *R* (version 4.0.0) [9] using ggplot2 package (version 3.3.1).

**Table 3:** Summary of percentages of available data over the observation period per laboratory parameter.

Parameter	Percentage of days with available data (%)
C-reactive protein	97
Platelet count	99
Prothrombin time	85
Fibrinogen	84
Factor II	82
Factor V	82
Factor VIII	85
D-dimers	84
Thrombin generation LT	58
Thrombin generation ttP	58
Thrombin generation pH	58
Thrombin generation ETP	58
Plasminogen activator inhibitor 1	84
Global fibrinolytic capacity	83
Antithrombin activity	84
Protein C activity	44
Free protein S antigen	44
Total TFPI	33
Free TFPI	33
Anti-Xa activity	82
von Willebrand factor antigen	21
von Willebrand factor activity	21
ADAMTS-13	22

LT, lag time; ttP, time to peak, pH, peak height; ETP, endogenous thrombin potential; TFPI, tissue factor pathway inhibitor.

## Ethics Statement

The observation was performed in accordance with the Declaration of Helsinki and after approval of the Ethics Committee of the CHU UCL Namur (NUB: B0392020000031).

## Declaration of Competing Interest

The authors declare that they have no known competing financial interests or personal relationships that have or could be perceived to have influenced the work reported in this article.
